# The Kaumoebavirus LCC10 Genome Reveals a Unique Gene Strand Bias among “Extended *Asfarviridae*”

**DOI:** 10.3390/v13020148

**Published:** 2021-01-20

**Authors:** Khalil Geballa-Koukoulas, Julien Andreani, Bernard La Scola, Guillaume Blanc

**Affiliations:** 1 MEPHI, APHM, IRD 198, Aix Marseille Université, Institut Hospitalo-Universitaire Méditerranée Infection, 13005 Marseille, France; khalil.geballa@mio.osupytheas.fr (K.G.-K.); miaguiabidou@gmail.com (J.A.); 2 Aix Marseille Université, Université de Toulon, Centre National de la Recherche Scientifique, Institut de Recherche pour le Développement, Mediterranean Institute of Oceanography UM 110, 13288 Marseille, France

**Keywords:** *Kaumoebavirus*, nucleo-cytoplasmic large DNA virus, *Nucleocytoviricota*, extended *Asfarviridae*, *Vermamoeba vermiformis*, gene strand bias

## Abstract

*Kaumoebavirus* infects the amoeba *Vermamoeba vermiformis* and has recently been described as a distant relative of the African swine fever virus. To characterize the diversity and evolution of this novel viral genus, we report here on the isolation and genome sequencing of a second strain of *Kaumoebavirus*, namely LCC10. Detailed analysis of the sequencing data suggested that its 362-Kb genome is linear with covalently closed hairpin termini, so that DNA forms a single continuous polynucleotide chain. Comparative genomic analysis indicated that although the two sequenced *Kaumoebavirus* strains share extensive gene collinearity, 180 predicted genes were either gained or lost in only one genome. As already observed in another distant relative, i.e., *Faustovirus*, which infects the same host, the center and extremities of the *Kaumoebavirus* genome exhibited a higher rate of sequence divergence and the major capsid protein gene was colonized by type-I introns. A possible role of the *Vermamoeba* host in the genesis of these evolutionary traits is hypothesized. The *Kaumoebavirus* genome exhibited a significant gene strand bias over the two-third of genome length, a feature not seen in the other members of the “extended *Asfarviridae*” clade. We suggest that this gene strand bias was induced by a putative single origin of DNA replication located near the genome extremity that imparted a selective force favoring the genes positioned on the leading strand.

## 1. Introduction

In 2003, the field of virology acquired a new dimension when the giant virus Mimivirus, infecting the amoeba *Acanthamoeba polyphaga*, was first described [1]. The approach to isolating viruses using *Acanthamoeba* cells as prey proved to be extremely prolific, as several highly diverse lineages of large and giant viruses were subsequently isolated from various environments due to their capacity to infect and multiply in this host. These include, for instance, *Marseillevirus, Pandoravirus, Mollivirus, Pithovirus, Medusavirus, Pacmanvirus*, etc., all belonging to the same broad group of Nucleo-Cytoplasmic Large DNA Viruses (NCLDV), a viral phylum recently renamed *Nucleocytoviricota* [2]. In 2015, attempts to diversify the host preys in co-culture assays led to the discovery of *Faustovirus* (FV) E12 infecting another amoebal species, i.e., *Vermamoeba vermiformis* (VV hereafter) [3]. Subsequently, this new VV host also proved to be very productive for the isolation of new large viruses. Not only were new specimens of FVs discovered [4,5,6,7], but also other more divergent lineages of *Nucleocytoviricota* such as *Kaumoebavirus* (KV) [8], *Orpheovirus* [9], *Tupanviruses* [10] and *Yasminevirus* [11]. Phylogenetic analysis of the only specimen of *Kaumoebavirus* (strain Sc, isolated in Saudi Arabia; referred to as KV-Sc hereafter) described so far placed this lineage at the root of the phylogenetic group formed by FV, *Pacmanvirus* (PV) and theAsfarviruses. This group forms one of the three main clades of *Nucleocytoviricota*, recently coined as the “Extended *Asfarviridae*” [12]. Thus, the KV lineage emerged at a strategic phylogenetic position for the understanding of this important viral clade, which includes the African swine fever virus (ASFV) [13]. This latter is the cause of African swine fever, an important disease that remains a serious threat to swine industries worldwide [14]. Although FV-E12 had a larger genome than KV-Sc (466 Kb vs. 351 Kb, respectively), both viruses encoded a similar number of genes (451 vs. 465 respectively). Both virions were icosahedral and devoid of fibrils; their capsids had similar reported sizes: 260 nm [15] and 250 nm [8] for KV and FV, respectively. The duration of the replicative cycle was about the same (16 H–20 H), including the intermediate steps (e.g., phagocytosis, membrane fusion, eclipse phase, virus factory, cell lysis). However, a notable difference in KV is the absence of morphological changes of the host nucleus, whereas in the FV infection cycle, a reorganization of the nucleus was observed after the eclipse phase. Here, we report the isolation and genomic analysis of a second isolate of KV, namely strain LCC10 (KV-LCC10 hereafter). KV-LCC10 was isolated from sewage water sampled in the south of France. This novel genomic sequence sheds new light on the genome structure, diversity and evolution within this viral lineage.

## 2. Materials and Methods

### 2.1. Sample Collection and Cultures

The KV-LCC10 strain was isolated from a sample of sewage water taken in La Ciotat, France on 1 November 2015. The sample was stored in a sterile tube at 4 °C in the dark for 4 days until its treatment and was part of the same sample batch in which *Orpheovirus* LCC2 was isolated [9]. The sewage sample was inoculated onto a monolayer of VV (strain CDC19) cells in starvation medium at 30 °C, as previously described in Reteno et al. [3]. Viral particles in the culture supernatant were serially diluted from 10^−1^ to 10^−11^ using the end-point dilution method. Briefly, the microplate was inoculated with each dilution in four wells that already contained rinsed *V. vermiformis* at a concentration of 10^6^ per mL. Amoeba lysis was monitored daily on the basis of inverted microscope observations. Only one well that simultaneously had a complete lysis at 30 °C in the highest dilution was used for virus production. Viral particles were purified as previously described [3]. Observation of KV-LCC10 using negative staining on electron microscopy [8] was done and captured on TECNAI G20 (FEI, Potsdam, Germany) at 200 keV. ImageJ was used to measure particle size.

### 2.2. Genome Sequencing and Analysis

Genome sequencing was performed on a MiSeq instrument, generating a total of 1,077,052 2 × 251 bp paired-end reads. AlienTrimmer was used for trimming and quality control of the raw reads with the following parameters *p* = 80, l = 100, and k = 10 [16]. The validated reads were then assembled by Spades v3.11.1 [17] using kmers of lengths 21, 33, 55, 77, 99 and 127. A large contig of 360,318 bp and another contig of 1032 bp with approximately twice the sequencing depth (X = 1591) of the large contig (X = 777) were obtained and analyzed in more detail. The reads aligning within the 500 bp at the extremities of both contigs were pooled and re-assembled separately. This allowed reconstruction of the junctions between the large contig and the 1032 bp contig, the latter being found to correspond to the sequence of two identical copies of terminal inverted repeats (TIR) near the extremities of the genome (hence the double coverage level compared to the large contig). In addition, a 102 bp small contig was generated, containing a sequence capable of folding into an incompletely base-paired hairpin structure according to Mfold [18]. Further analysis of the paired reads jointly aligned with both the TIR and the 102 bp sequences revealed how these structures were contiguous in the genome. We found that the hairpin sequence was joined at both its 5′ and 3′ ends with either the forward and reverse strands of the outer end of the TIR sequence. These observed junctions are compatible with the hypothesis that KV-LCC10 has a linear genome with covalently closed hairpin termini flanking the TIRs. Furthermore, the hairpin sequences coexisted in inverted and complementary forms, similar to the flip/flop isoforms of the terminal hairpin sequences of the Poxvirus genomes [19].

Open Reading Frames (ORF) were predicted using GenemarkS (option—virus) [20]. In addition, ORFs were extracted in the intergenic sequences defined by GenemarkS to recover genes potentially missed in the first round. ORFs shorter than 300 bp were further examined by a blast search again the NR protein database. Only ORFs <300 bp that had a significant match (E-values < 1 × 10^−5^, excluding match to KV-Sc) were conserved in the annotation. Potential tRNA genes were searched using ARAGORN [21] and tRNAscan-SE–online version [22]. The structure of the Major Capsid Protein (MCP) gene and the RNA polymerase subunit gene, both of which contained introns, was determined manually by searching genomic regions (i.e., exons) of high similarity with the corresponding PV proteins (whose genes do not contain introns) using TBLASTN. The boundaries between exons and introns were determined based on the returned alignments. Protein families between viruses were constructed using OrthoFinder [23]. For the sake of consistency, the KV-Sc genome was re-annotated using the same procedure as described above for comparative analysis. We predicted 456 ORFs with this annotation procedure versus 429 annotated genes in the GenBank entry for KV-Sc (KX552040). Functional annotation of the predicted proteins was performed by combining information from BLASTP similarity searches against SwissProt and Uniprot, as well as well as similarity search against protein motifs databases using PfamScan [24] and Interporscan [25]. The genes that did not have any detectable homologs in other organisms or viruses were annotated as hypothetical proteins. The complete genome annotation of KV-LCC10 was submitted to the Genbank database under the accession number MT334784. Multiple alignments of protein families were generated with MAFFT [26], after which positions containing more than 90% gaps were removed from the alignment. Phylogenetic trees were reconstructed using FastTree [27] with default parameters.

The genome-wide CDS skew was calculated by subtracting the cumulative length of the coding sequences on the reverse strand to the cumulative length of the coding sequences on the forward strand. To make CDS skew values comparable between genomes, these were normalized by the total length of the genome’s coding sequences. Statistical analysis of the genome-wide CDS skew was performed by randomly reallocating the virus’ genes on the two strands and calculating the randomized-genome-wide CDS skew. This procedure was repeated 1000 times. The mean and standard deviation of the 1000 randomized-genome CDS skew values were obtained and a Z-score was calculated as the difference between the real genome-wide CDS skew and the mean randomized genome-wide CDS skew, divided by the standard deviation. The Z-score was compared to the normal distribution (i.e., Z-test) to determine the *p*-value associated with the null hypothesis that the real genome-wide CDS skew is compatible to a random distribution of genes between the two strands.

## 3. Results and Discussion

### 3.1. Isolation and Electron Microscopy

**Figure 1:**
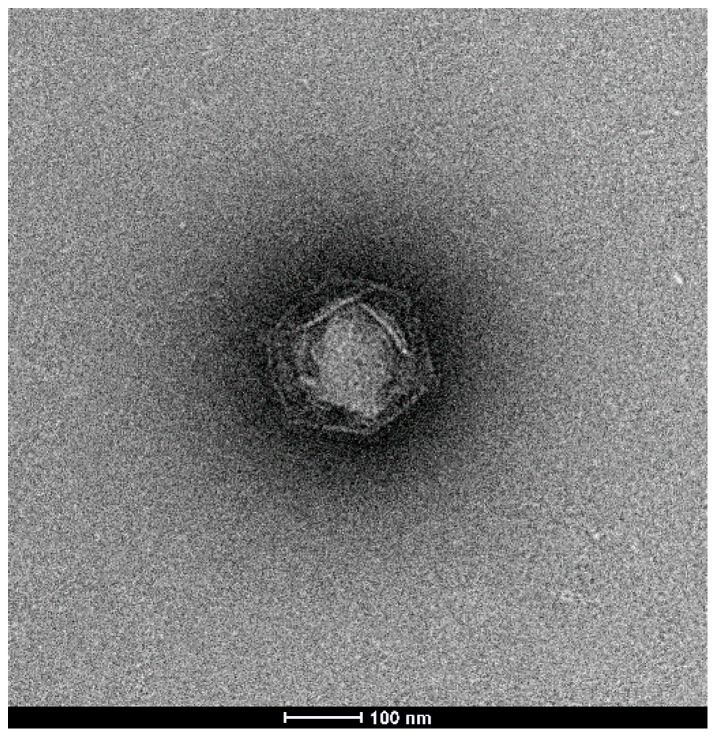
Electronic microscopy observation of negatively stained Kaumoebavirus LCC10.

Co-culture of *Vermamoeba vermiformis* with a sewage water sample taken in La Ciotat, France revealed a lytic agent of the amoebal cells, which was cloned by further serial dilution steps. We performed negative staining on electron microscopy and observed an icosahedral virus of about 240 nm (Figure 1). Subsequent genome sequence analysis will reveal that this virus is related but not identical to the KV-Sc. However, microscopy observations did not reveal obvious differences with KV-Sc both in terms of virion shape and size of particles. The length of the replication cycle was also not noticeably different (16 H–20 H).

### 3.2. KV Genome Content

**Figure 2:**
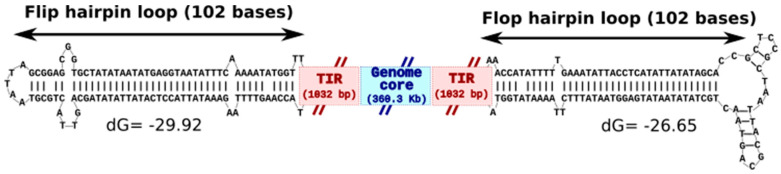
Sequence structures at the ends of the KV-LCC10 chromosome. Schematic representation of the linear KV-LCC10 genome with covalently closed, incompletely base-paired hairpin termini flanked by TIRs. The 102 bp hairpin sequences and TIRs on each side of the genome are inverted and complementary. The minimum free energy (dG) fold of the two versions of the hairpin loop (flip/flop) was inferred using the MFOLD program. Please note that the positioning of the flip/flop versions relative to the left/right sides of the genome could not be determined using the current data and is therefore represented here arbitrarily.

The KV-LCC10 genome was assembled into a 362.6-kb contig, with each end terminated by a 102-bp incompletely base-paired hairpin loop immediately adjacent to a 1032-bp TIR inward (Figure 2). The GC content was 43.1%, which is only slightly lower than the 43.7% of the KV-Sc genome (Table 1). The 102 bp hairpin sequences on each side of the genome are inverted and complementary (known as flip/flop isoforms [19]). Furthermore, both the 5′ and 3′ hairpin extremities are joined to either the forward or reverse strand of the TIR regions indicating that the KV-LCC10 genome is a single contiguous polynucleotide chain which self-anneals into a linear duplex with covalently closed hairpin termini. The structure of the hairpin loops with the lowest free energy differed at their apex owing to a few differences in nucleotide complementarity between flip/flop versions. Similar chromosome architectures comprising covalently linked extremities of the linear DNA genome have been described in other NCLDVs such as poxviruses [19], chloroviruses [28] and the more closely related ASFVs [29]. The existence of terminal hairpin loops has not been specifically verified in the other extended Asfarviridae, but it is possible that similar structures will be eventually discovered at the extremities of the FVs and PV genomes. In the genomes of Poxviruses, sequences adjacent to the hairpin loops are essential for both replication and resolution of the concatemeric replication intermediates [30,31]. Experimental studies will be needed to validate whether this is also the case for KV-LCC10.

**Figure 3:**
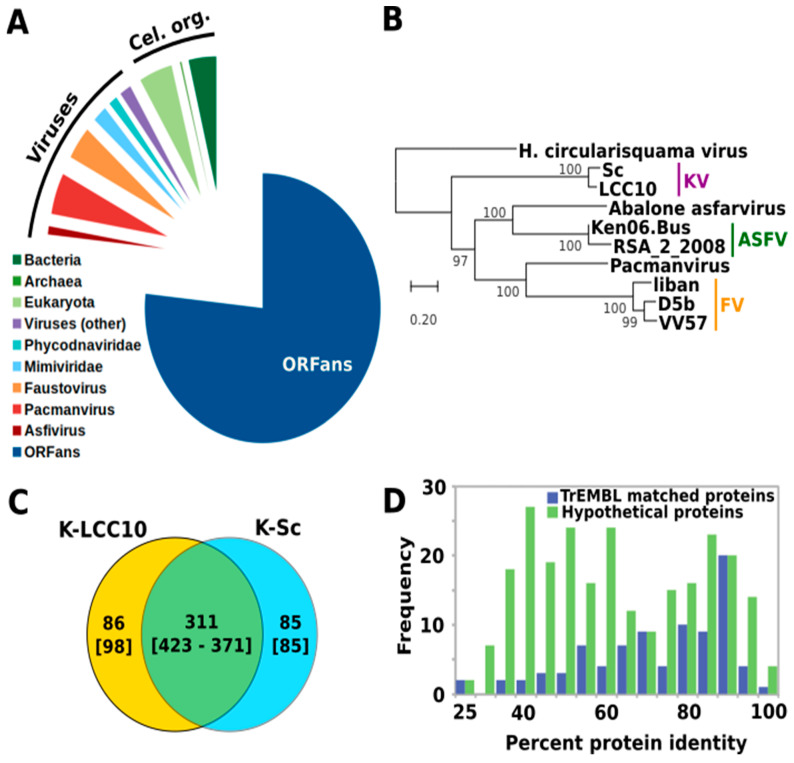
Comparative genomics of KV-LCC10. (**A**) Taxonomic classification of the KV-LCC10 protein best matches in the TrEMBL database. Best matches were identified with MMSEQS and a E-value threshold of 1E-05. Match to KV-Sc proteins were not included. (**B**) Phylogenetic tree of the DNA polymerase proteins. The multiple alignment was done with MAFFT and the phylogenetic tree was reconstructed with FastTree using default parameters. Branch supports indicated beside internal nodes were obtained using the SH-aLRT method as implemented in FastTree. The scale bar indicates the number of substitutions per amino acid sites. (**C**) Protein families shared between the two KVs strains. Numbers outside and inside brackets indicate the number of protein families involved in the category and the cumulative number of proteins in those families, respectively. For families shared between K-LCC10 and K-Sc, 423 is the number of proteins for K-LCC10 and 371 is the number of proteins for K-Sc. (**D**) Protein sequence similarity between reciprocal best matches between the two KV strains. The blue distribution represents proteins that have a significant match in the TrEMBL database (with exclusion of KV entries); the green distribution represents proteins with no significant matches in TrEMBL and therefore considered as hypothetical proteins.

Sequence annotation revealed 507 predicted protein genes, 76% (*n* = 385) of which had no detectable homologs in viruses outside of the KVs themselves or in cellular organisms and were annotated as ORFans encoding hypothetical proteins (Figure 3A). Seventy-six protein genes had a best match in viruses (NCLDV exclusively), the majority of which were in FVs (*n* = 20) and PV (*n* = 25). 41 additional genes had their best match in cellular organisms, distributed approximately equally between eukaryotes (*n* = 23) and prokaryotes (*n* = 20). Phylogenetic reconstruction using the predicted DNA polymerase protein as marker placed KV-LCC10 and KV-Sc at the root of the Asfarvirus-Pacmanvirus-Faustovirus cluster (Figure 3B). The average pairwise protein distance between 40 single-copy core proteins conserved across all extended-Asfarviridae (Table S1) suggests that sequence divergence between KV-LCC10 and KV-Sc (*d* = 0.11) is comparable to the sequence divergence between clades D and E9 of Faustovirus (*d* = 0.11) described in Geballa-Koukoulas et al. [7]. Although KV-LCC10 shared 84% (*n* = 423) of its genes with KV-Sc (Figure 3C), its genome was 11 Kb larger and encoded 50 more predicted genes than KV-Sc in total. KV-LCC10 had 98 proteins with no homologue in KV-Sc whereas KV-Sc had 82 proteins without homologue in KV-LCC10. KV-LCC10 unique proteins included KLCC10_0094, a protein containing a putative eukaryotic-type phosphorylated CTD interacting factor 1 WW domain which may be involved in mRNA synthesis by modulating RNAP IIO activity [32]. No homologue was found in other viruses suggesting that the gene has been acquired by lateral gene transfer from a eukaryotic host. Two other LCC10-specific proteins (KLCC10_0184 and KLCC10_0264) had homologues in FVs but no functional attributes could be assigned to them. The corresponding genes were likely inherited from the common ancestor of FVs and KVs, but were subsequently lost in the KV-Sc lineage. The remaining KV-LCC10 specific proteins were all hypothetical proteins. In contrast, 15 KV-Sc genes absent in LCC10 had homologues in related viruses suggesting that these genes were specifically lost in the KV-LCC10 lineage. The encoded proteins included a component of a putative fatty acid hydroxylase, 2 transposases, a radical SAM enzyme and an endonuclease. In addition, 2 KV-Sc specific proteins were similar to a eukaryotic TNF receptor-associated factor family protein and a prokaryotic deoxyribodipyrimidine photolyase-related protein, which might have been acquired by lateral gene transfer from cellular organisms. Search for duplicated genes showed that KV-LCC10 had 57 more paralogous genes than KV-Sc (i.e., 183 vs. 116 for KV-LCC10 and KV-Sc respectively). Thus, the difference in genome length between the two strains is mostly explained by extra gene duplications in KV-LCC10.

The protein identity between KV best reciprocal hits ranged from 23% to 97%, with a mean value of 63% (Figure 3D). The distribution of sequence identities for the hypothetical proteins showed two modes: a first mode between 70% and 100% sequence identity which overlapped with the single mode of the distribution for proteins with a database match. The second mode corresponded to an excess of protein pairs with lower sequence identities (35–65%). This difference between the two distributions suggests that a fraction of the proteins uniquely found in KV tends to diverge more rapidly than KV proteins conserved in other viruses or organisms. Alternatively, a fraction of the KV-specific proteins could result from gene over-prediction (i.e., a predicted ORF in an intergenic sequence that evolved under no selective pressure). KV-LCC10 proteins with a database match had a mean length of 412.0 amino acids (aa), whereas hypothetical proteins had a mean length of 167.7 aa. The length distributions of hypothetical proteins did not differ ostensibly between those conserved with >60% similarity (mode 1) or <60% similarity (mode 2) with their KV-Sc orthologue (Figure S1A). This indicates that short genes were not specifically confined to the category for which in silico predictions are a priori the most uncertain (i.e., hypothetical protein genes that exhibit low conservation between KV strains). In addition, no marked difference on average amino acid composition was observed between KV-LCC10 proteins at different conservation levels (Figure S1B), whereas this would be expected if one of the categories contained a large number of proteins derived from false predictions. Altogether, these results provide no evidence for a high rate of gene over-prediction in KV-LCC10.

**Figure 4:**
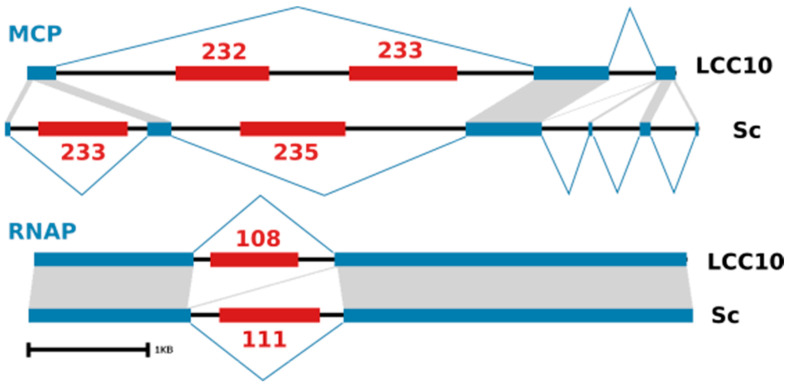
Type-1 introns in KV genes. Schematic representation of the MCP and RNAP genes in the two KV strains (KV-LCC10 above, KV-Sc below). Exons are represented by blue rectangles connected by blue lines showing splicing sites. Nucleotide sequence similarity is indicated by grey shaded areas. Homing endonuclease ORFs are shown by red rectangles, with numbers referring to the gene locus id in the respective Genbank records.

The MCP gene and the RNA polymerase subunit 1 gene were found to contain type I introns in both KV strains (Figure 4). Some of these introns contained one or two ORFs encoding a GIY-YIG family endonuclease, which may have been involved in the insertion of the introns themselves. With the exception of the first and second introns of the KV-LCC10 and KV-Sc MCP genes respectively, the introns in the two viral strains were located at non-orthologous positions. Furthermore, their sequences shared no detectable nucleotide similarity. However, they tended to accumulate in specific regions of the genes, as is the case for the RNAP introns or the second and third + fourth introns of the MCP KV-LCC10 and KV-Sc genes respectively, therefore suggesting the existence of intron insertion hotspots. The phylogenetic tree of intron-encoded endonucleases showed no evidence of orthologous relationships between the KV proteins (Figure S2)—normally visible as a clade containing one protein from each strain—suggesting that their divergence predates the last common ancestor of KV. Thus, taken together, our results indicate that the intron/exon structure of the MCP and RNAP1 genes in KVs results from an intron loss-and-gain mechanism that operates since the separation of the two lineages, possibly mediated by the intron-encoded homing endonucleases.

### 3.3. KV Genome Organization

**Figure 5:**
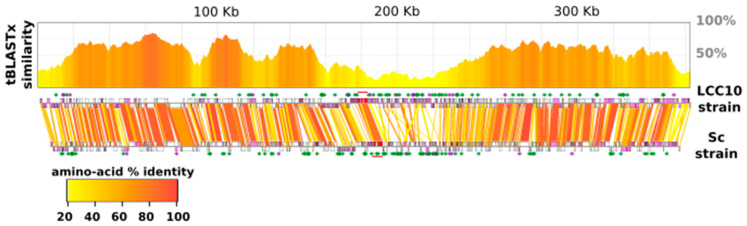
Genome conservation and colinearity between KV strains. Top panel: Levels of amino-acid similarity between the LCC10 and Sc genomes as measured by the tBLASTx program. The genomic coordinates refer to LCC10 strain. Bottom panel: gene collinearity between the LCC10 (top) and Sc (below) genomes. Open and purple-filled boxes represent single copy genes and genes engaged in a multi-gene family, respectively. Green asterisks indicate genes that are unique to the considered KV strain (i.e., without a BLASTP match with e-value < 1 × 10^−5^ against the other KV strain), whereas purple asterisks show strain specific genes that are also engaged in a multi-gene family. Colored links associate the putative LCC10 and Sc orthologous genes identified by the reciprocal best BLAST hit criterion. The coloring of the links represents the level of amino-acid similarity between orthologous proteins according to the color scale given. The horizontal red lines mark the position of the MCP genes.

Comparison of the gene orders along the chromosomes indicated that the two KV strains retained extensive genome collinearity with only a very few possible gene rearrangements in the middle of the chromosome and its extremities (Figure 5). TBLASTx measures of the level of amino-acid similarity along the chromosomes revealed that these same rearranged regions were globally more divergent than the inside of the two arms. These regions were also enriched in KV-LCC10-specific genes and contained a higher proportion of duplicated genes (Figure S3A). In contrast, the inside of the chromosome arms was enriched with genes of viral origin and single-copy genes while the center of the chromosome contained a high frequency of genes whose best match belonged to cellular organisms (Figure S3B). Altogether, these observations pointed to a higher rate of evolution in the middle and ends of the chromosome of the KV genomes, driven by sequence duplication, accumulation of strain specific genes and genes of possible horizontal origins, as well as intron gain and loss (i.e., the central region contained the MCP gene).

### 3.4. KVs Genome Has a Unique Gene Strand Bias

When analyzing the structure of the KV-LCC10 genome, we noticed a bias in the distribution of genes on both DNA strands of the chromosome (Table 2). Indeed, the forward strand (i.e., arbitrarily defined according to the orientation of the genome sequence recorded in GenBank) carried 311 predicted genes while the reverse strand only carried 196. This represents an excess of 58 genes (i.e., 11.3% of the genome repertoire) on the positive strand compared to what would be expected if the genes were distributed equitably between the 2 strands. Although the reported bias is only modest, the probability of obtaining such a difference in a model of random distribution of genes between the two strands is *p* = 2 × 10^−7^ according to the binomial law. This gene strand bias was further characterized by calculating the genome CDS skew, which measures the total excess of coding sequences on the forward DNA strand. A genome CDS skew corresponding to 26.4% of the total coding sequence content was found on the positive strand, a very unlikely figure if genes were randomly distributed between the two strands (*p* = 9 × 10^−5^ according to the Z-score test; see methods). To rule out the possibility that the observed bias was due to an effect of gene over-prediction, we reanalyzed the genome after discarding genes with no detectable homologues in sequence databases (i.e., genes coding for a hypothetical protein). The gene strand bias was still significant: 74 genes versus 46 genes on the forward and reverse strands respectively, corresponding to a gene excess of 11.7% on the forward strand (*p* = 7 × 10^−10^). We also considered the hypothesis that the bias was due to tandem gene duplications, which results in the multiplication of genes on the same strand. After eliminating extra copies of tandemly arrayed paralogues, we still observed a significant difference in the number of genes carried by the two strands (10% gene excess, *p* = 9 × 10^−6^).

Then, we investigated whether a similar gene strand bias existed in the viruses related to KV-LCC10. Not surprisingly a similar bias was observed in KV-Sc owing to the high level of genome conservation with KV-LCC10. This bias was measured regardless of the version of the annotation considered (previous genbank annotation stand excess = 11.5%, *p* = 1 × 10^−6^; our reannotation strand excess = 11.4%, *p* = 6.4 × 10^−7^). In contrast, the genomes of ASFV, FV and PV only showed minor biases, between −2% and 4% of excess genes on the positive strand. The associated probabilities were either not significant (ASFV, *p* = 0.34) or at most borderline significant (FV and PV, *p* = 0.03). This suggests that a biased organization of genes settled specifically in the KV lineage or existed in the ancestor of KV, ASFV, FV, and PV but was subsequently lost in the lineage leading to ASFV, FV, and PV.

**Figure 6:**
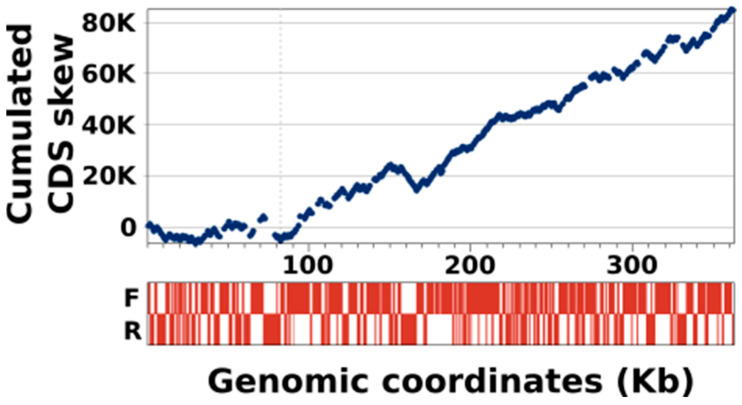
Cumulative CDS skew of the KV-LCC10 genome. The top panel shows the evolution of the cumulative CDS skew curve along the genome. The horizontal dashed line marks the position of the minimum value of the cumulative skew, near the coordinate 82Kb. The bottom panel shows the distribution of the coding sequence (in red) on the forward (F; upper lane) and reverse (R; lower lane) strands.

We then investigated whether this strand bias affected the entire KV-LCC10 genome or only specific regions. To this end, we generated the cumulative CDS skew curve (CSCC hereafter) shown in Figure 6, which fluctuates along the genome sequence according to the excess of coding sequence between the two DNA strands. The region extending from the left end of the chromosome to position 82Kb was characterized by a flat CSCC, indicating that the coding sequences are roughly equally distributed between the two strands in this segment of the genome. In contrast, from position 82 Kb to the right end of the chromosome, which represents more than ¾ of the genome (77%), the CSCC showed a generally increasing trend, with only a few brief minor reversals. This trend reflects a significant excess of coding sequence on the forwards strand throughout this segment of the chromosome.

Therefore, it appears likely that the preferential distribution of the genes on the positive strand over an extended region of the chromosome is the result of a biased process or a specific selective pressure that has led to the organization of the KV genome as we have observed it. However, this hypothetical process or pressure is presently unknown. Genes in bacterial genomes tend to be carried by the leading strand presumably to minimize the conflicts between processing DNA and RNA polymerase complexes. The resulting gene organization is reflected in a characteristic V-shape of the CSCC, defining two regions with opposite gene strand bias [33]. The inflection points in the CSCC are thought to correspond to the sites of origin and termination of DNA replication. In large DNA viruses, the link between DNA replication and gene orientation is not that well established. In bacteriophage T4; however, the genome exhibits regions in which gene orientation is strongly biased [34]. Although the majority of the replication initiation events is mediated by re-combinational intermediates which presumably take place at any position in the genome (recombination-dependent DNA replication), bacteriophage T4 contains several experimentally validated replication origins which are only used in the first rounds of replication [35]. The boundaries of some of the regions with a high gene strand bias appear to coincide with the location of some of the replication origins [34], suggesting that DNA replication also influences the orientation of genes to some extent in this virus. Poxviruses DNA replication is certainly the best studied among NCLDVs; however, its mechanism is still uncertain. Two models of genome replication have been proposed, one based on the rolling-hairpin mechanism involves only leading-strand DNA synthesis starting at a nick on one strand [36,37], the other involving a replication fork with leading- and lagging-strand synthesis [38]. In both models the initiation of replication is thought to occur near the terminal hairpin loops, which has been supported by experimental data [38,39]. By extrapolation of the findings made in these model viruses, we can hypothesize that a single replication origin is located near the left-side hairpin loop of the KV-LCC10 genome. The fact that the KV-LCC10 genome encodes a primase-helicase and DNA ligase suggests that its DNA replication is RNA-primed and involves a replication fork with leading- and lagging-strand synthesis. This model of DNA replication might imprint a selective force favoring the positioning of genes on the leading strand, which would explain the specific gene strand bias observed in KV genomes.

## 4. Conclusions

In 2015, the shift from *Acanthamoeba sp.* to *Vermamoeba vermifomis* as amoebal prey in co-culture assays fueled the discovery a variety of novel NCLDV lineages, including KVs. KV appears as the most basal lineage of the extended Asfarviridae clade and therefore is phylogenetically equidistant from its other viral members. However, features of genome evolution in KV are more similar to FV than to the other member viruses. Indeed, both KV and FV exhibited more divergent chromosome center and ends, as well as a remarkable variability in the distribution of introns in the MCP gene [7]. In contrast, such an organization is not observed in the ASFV genome, and the MCP genes of the ASFV, the Abalone “asfarvirus” and PV are devoid of introns. These latter viruses infect hosts distinct of VV (i.e., pigs, abalone and Acanthamoeba sp., respectively). It is unclear whether the common traits of genome evolution between KV and FV originate from of the common ancestor of the extended Asfarviridae or whether they result from a convergent evolution induced by the VV host. This hypothesis will be testable when the genome sequences of new strains of VV infecting viruses outside of the extended Asfarviridae clade (i.e., Orpheovirus, Tupanviruses, and Yasminevirus) are available for comparative genomic study.

## Figures and Tables

**Table 1:** Genomic features of KVs.

Strain	LCC10	Sc
Genome length	362,586	350,731
GC%	43.1	43.7
TIRs (bp)	1032	1407
Predicted Genes (Gene families)	507	456
Hypothetical proteins	385 (76%)	304 (67%)
Paralogs (Gene families)	183 (44)	116 (35)
Strain-specific genes	98	82

**Table 2:** Gene strand bias in KV and virus relatives.

Species	Accession Number	Gene Number	Strand Excess (a)	*p*-Val (b)	CDS Cumul. Length	Genome CDS Skew	*p*-Val (c)
Kaumoebavirus LCC10 (KV-LCC10)	MT334784	507	11.3%	2 × 10^−7^	342,156	26%	9 × 10^−5^
w/o hypothetical protein genes		120	11.7%	7 × 10^−3^	152,698	34%	2 × 10^−3^
w/o tandemly duplicated genes		463	10%	9 × 10^−6^	316,152	24%	3 × 10^−4^
Kaumoebavirus Sc (KV-Sc)	KX552040	429	11.5%	1 × 10^−6^	280,782	29%	2 × 10^−5^
African swine fever virus (ASFV)	ASU18466	152	−2%	0.34	150,903	−15%	0.19
Faustovirus (FV)	MN830295	503	4%	0.03	439,093	9%	0.19
Pacmanvirus (PV)	LT706986	465	4%	0.03	353,586	5%	0.29

(a) Percentage of the gene content in excess on the forward strand; (b) According to the bionomial test; (c) According to the Z-score test.

## Data Availability

The complete genome annotation of KV-LCC10 is openly available in the Genbank database under the accession number MT334784.

## Supplementary Materials

The following are available online at https://www.mdpi.com/1999-4915/13/2/148/s1, Figure S1: Length distribution and amino acid composition of KV-LCC10 proteins. Figure S2: Phylogenetic reconstruction of homing endonucleases, Figure S3: gene distribution in the K-LCC10 genome. Table S1: average pairwise protein distances between 40 single-copy core gene families of extended *Asfarviridae.*
