# Supplementary material for: The Kaumoebavirus LCC10 Genome Reveals a Unique Gene Strand Bias among “Extended Asfarviridae”
Source: Viruses. 2021 Jan 20;13(2):148. doi: 10.3390/v13020148 (PMC7909422; doi:10.3390/v13020148)
Supplement: Supplementary file 1 [file viruses-13-00148-s001.pdf]

A

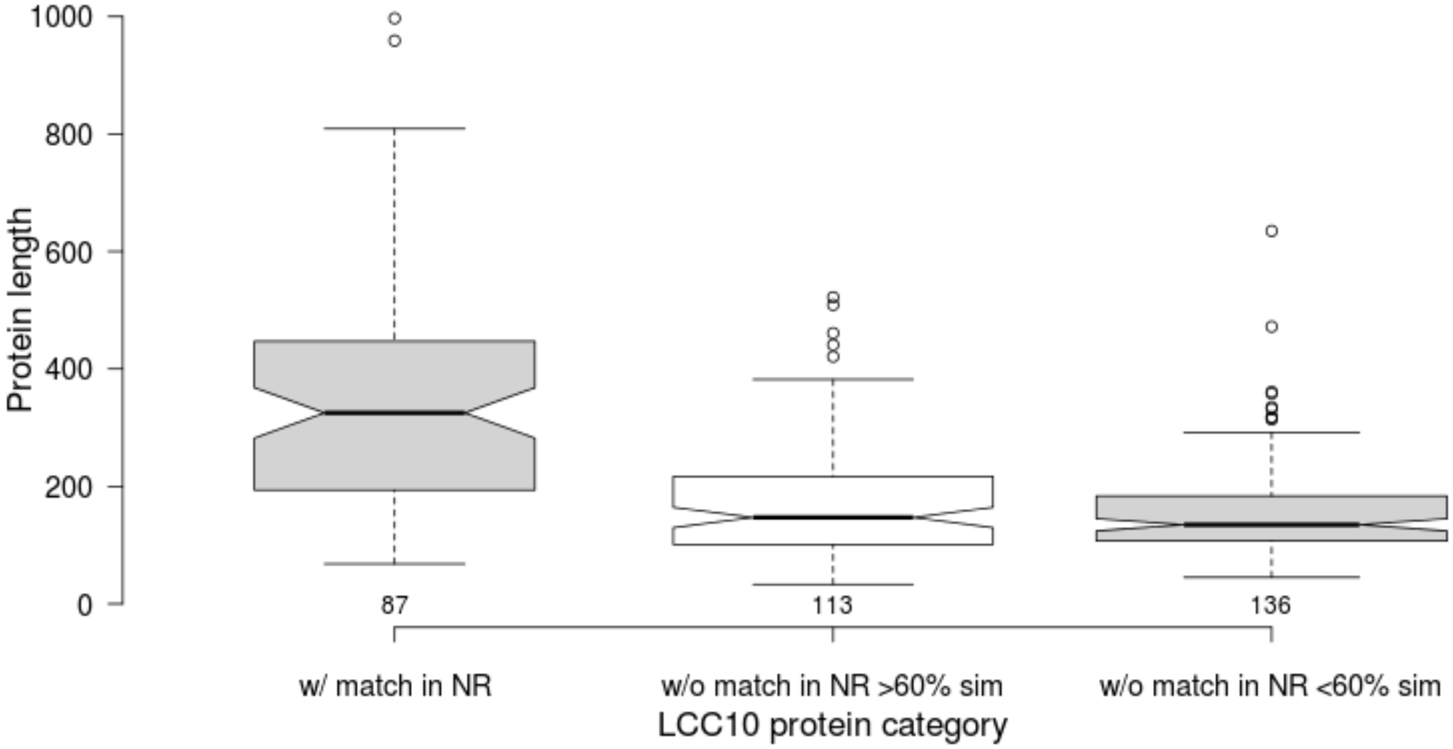

B

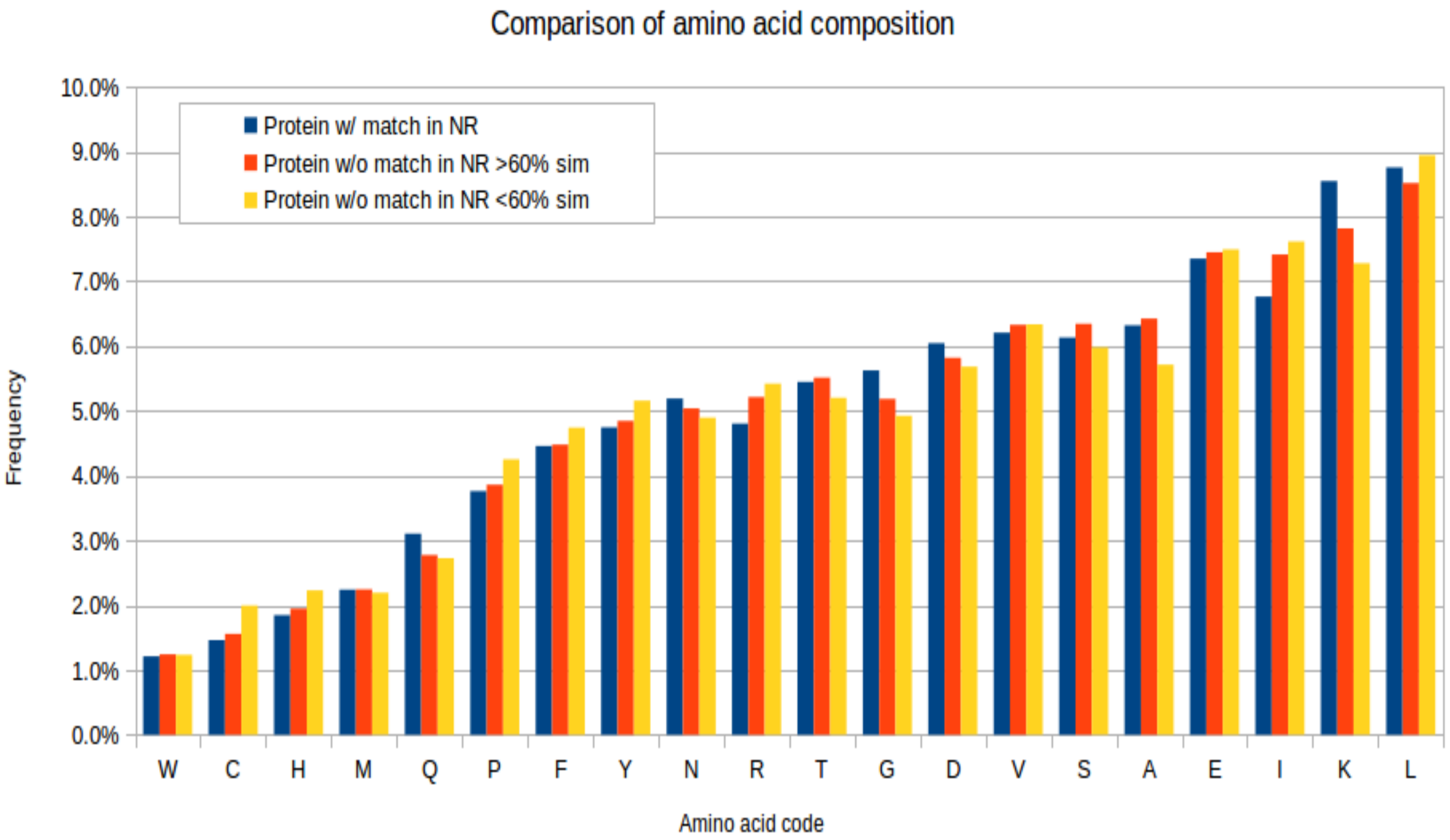

Figure S1: Length distribution and amino acid composition of KV-LCC10 proteins (A) Boxplots representing the length distribution of 83 proteins with a match in the NR database and a identified orthologue in KV-Sc, 113 hypothetical proteins with >60% identity with their KV-Sc orthologue and 136 hypothetical proteins with <60% identity with their KV-Sc orthologue. (B) Amino acid frequencies in the same 3 protein categories.

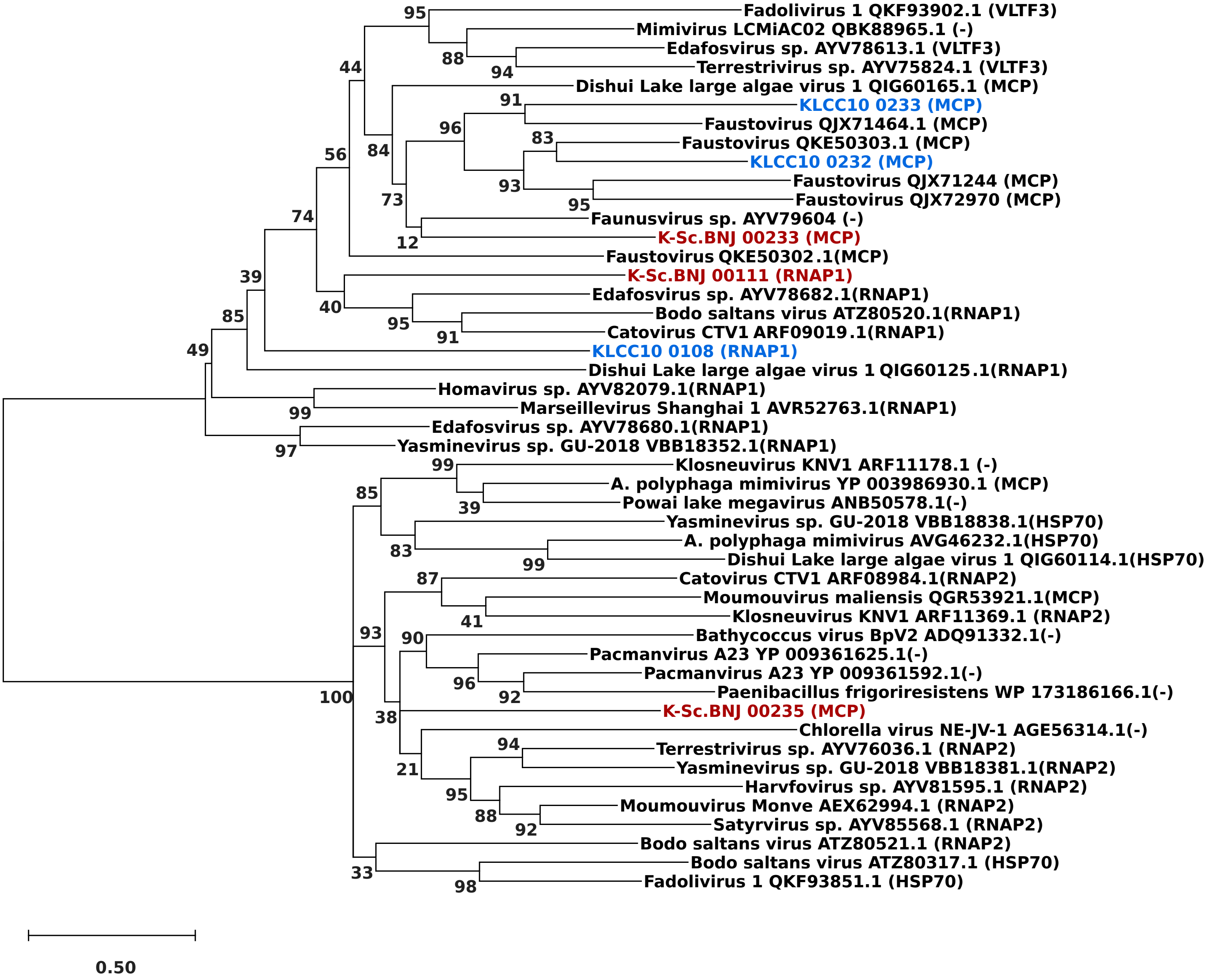

Figure S2. phylogenetic reconstruction of homing endonucleases. The phylogenetic tree was reconstructed with FastTree using its default parameters. The SH-aLRT branch support is indicated beside each internal node. The scale bar represents the number of amino-acid substitution per site. The name of the gene haboring the intron-encoded nuclease resides is given between parentheses. (-) indicates that the endonuclease ORF is not inserted in an intron.

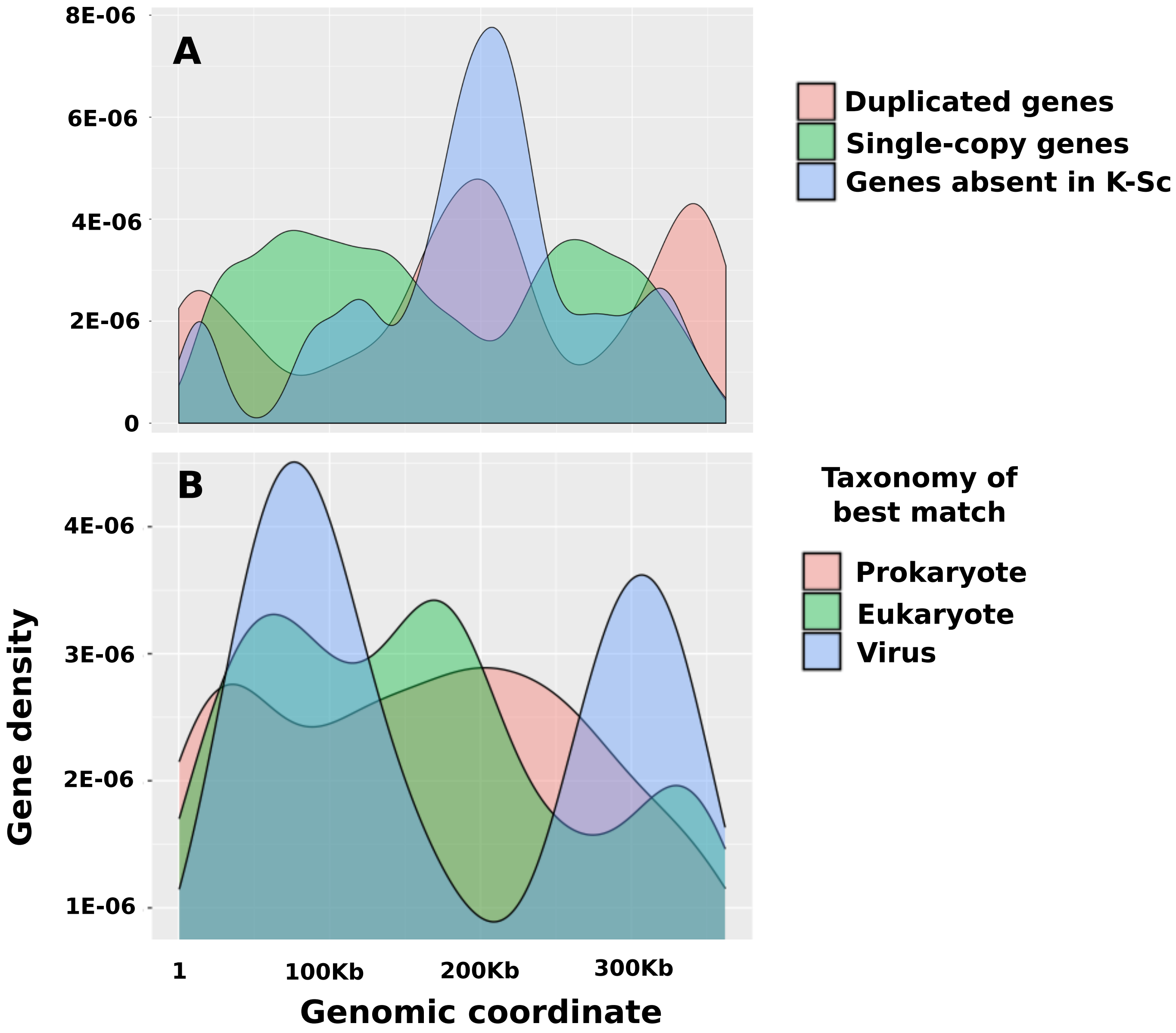

Fig. S3: Gene distribution in the K-LCC10 genome
(A) Density distributions for duplicated genes, single-copy genes and genes not found in the K-Sc genome. (B) Density distributions according to the taxonomy of the gene best match in TrEMBL.

Table S1 : average pairwise protein distances between 40 single-copy core gene families of extended *Asfarviridae*

	Faustovirus M6 (Clade E9)	Faustovirus D3 (clade D)	Faustovirus E12 (Clade M/L)	Pacmanvirus A23	Pacmanvirus A19	ASFV Ken06.Bus	ASFV Odintsovo_02.14	Kaumoebavirus LCC10
Faustovirus D3 (clade D)	0,12							
Faustovirus E12 (Clade M/L)	0,21	0,21						
Pacmanvirus A23	1,15	1,14	1,13					
Pacmanvirus A19	1,15	1,14	1,13	0,04				
ASFV Ken06.Bus	1,67	1,67	1,68	1,52	1,52			
ASFV Odintsovo_02.14	1,67	1,67	1,68	1,52	1,52	0,04		
Kaumoebavirus LCC10	1,73	1,72	1,73	1,61	1,62	1,76	1,76	
Kaumoebavirus Sc	1,74	1,73	1,74	1,62	1,62	1,77	1,77	0,11

Information : 40 single-copy core protein families were identified in 33 fully sequenced extended-Asfarviridae (16 Fautoviruses, 13 Asfarviruses, 2 Pacmanviruses, 2 Kaumoebaviruses) using OrthoFinder. A multiple alignment of each protein family was generated with MAFFT. After removing columns with gaps, the 40 multiple alignments were concatenated into a single one containing 15,329 amino acid sites available for pairwise distance calculation. Pairwise amino acid distances were calculated using the codeml program and the WAG subsitution model. This table only showcases 3 Fautovirus representatives of clades E9, D and M/L and 2 ASFV representatives that had the largest protein distances between all ASFV genomes.
